# Comparative Effects of Different Surface Treatments on Shear Bond Strength of Metal Brackets Bonded to Eroded Enamel: An in Vitro Study -

**DOI:** 10.31661/gmj.v14iSP1.3951

**Published:** 2025-12-15

**Authors:** Milad Soleimani, Hengameh Banaei, Manijeh Mohammadian

**Affiliations:** ^1^ Department of Orthodontics, School of Dentistry, Shahid Beheshti University of Medical Sciences, Tehran, Iran; ^2^ Student Research Committee, Alborz University of Medical Sciences, Karaj, Iran; ^3^ Department of Dental Biomaterials, School of Dentistry, Iran University of Medical Sciences, Tehran, Iran

**Keywords:** Lasers, Solid-State, Orthodontic Brackets, Shear Strength, Tooth Erosion

## Abstract

**Background:**

This study aimed to compare the effects of different surface treatments on shear bond strength (SBS) of metal brackets to eroded enamel.

**Materials and Methods:**

In this in vitro study, 76 extracted premolars were immersed in Coca-Cola 4 times, each time for 2 minutes to cause enamel erosion. They were then randomly assigned to 4 groups (n=19) for surface treatment by acid etching (control), bur grinding plus acid etching, sandblasting plus acid etching, and erbium-doped yttrium aluminum garnet (Er:YAG) laser irradiation plus acid etching. Metal brackets were then bonded to the buccal surface of the teeth and after thermocycling, their SBS was measured in a universal testing machine. After debonding, the adhesive remnant index (ARI) score was determined under a stereomicroscope. SBS of higher than 6 was considered as optimal (Reynolds threshold).

**Results:**

The control group showed the highest, and the laser group showed the lowest SBS; however, the difference in SBS was not statistically significant among the four groups (P=0.35). Acid etching group had 2 cases of failure in SBS values, while other groups had none. The study groups had no significant difference in the ARI scores either (P=0.82); nonetheless, sandblasting and laser groups had the highest frequency of ARI score 3 (all adhesive remaining on the surface).

**Conclusion:**

Bur grinding, sandblasting, and Er:YAG laser irradiation did not significantly change the SBS of metal brackets to eroded enamel compared with acid etching alone, and all the tested methods yielded acceptable SBS values.

## Introduction

In orthodontic treatment, brackets are bonded to the enamel surface. However, the enamel surface is not sound and intact in all cases, and may be hypoplastic, eroded, or fluorosed, making it difficult to achieve an optimal bracket bond strength [1]. Among these conditions, enamel erosion poses a significant challenge due to its impact on the enamel's integrity. Enamel erosion is defined as irreversible demineralization of the enamel surface due to the effect of acidic chemical agents. Normally, exposure of enamel to acidic agents results in its temporary demineralization, and the buffering capacity of the saliva changes the pH of the oral environment and remineralizes the enamel surface [2][3]. However, if the acidity exceeds the buffering capacity of the saliva (due to high frequency of exposures or excessively low pH), remineralization does not occur, and the enamel remains irreversibly demineralized [2][3]. Approximately 30% of the population suffer from dental erosion [4][5]. Xerostomia, aging, and poor socioeconomic status contribute to dental erosion [6]. Eroded enamel is more susceptible to mechanical stress since it has lost a portion of its mineral content due to acid exposure. Resultantly, brackets often have a lower bond strength to eroded enamel compared with sound enamel [5][7][8][9][10].

By the increasing demand for orthodontic treatment among adults, and increased prevalence of dental erosion with age, bracket bonding to eroded enamel has become a challenge for orthodontists [4][5]. Therefore, researchers have been in search of strategies to increase the shear bond strength (SBS) of brackets to eroded enamel. It has been reported that acid etching of an eroded enamel surface alone for bracket bonding decreases the enamel microhardness and strength. Therefore, surface treatments such as sandblasting [9], application of sodium calcium silicate [5], TiF4 varnish [8], or casein phosphopeptide amorphous calcium fluoride paste, CO2 laser irradiation [10], and bur grinding have been suggested to increase the microhardness of the eroded enamel, and subsequently enhance bracket SBS. Sandblasting, bur grinding, and laser irradiation increase enamel porosities and surface roughness, which are believed to increase adhesive penetration, and improve the bond strength [11].

Considering the lower microhardness of eroded enamel compared with sound enamel [5][7][8][9][10], and the challenges encountered in bracket bonding to eroded enamel, this study aimed to compare the effects of different surface treatments including acid etching, bur grinding, sandblasting, and erbium-doped yttrium aluminum garnet (Er:YAG) laser irradiation on SBS of metal brackets to eroded enamel. The null hypothesis of the study was that no significant difference would be found among the aforementioned four surface treatments with respect to their effect on SBS of metal brackets to eroded enamel.

## Materials and Methods

This in vitro experimental study was conducted on 76 sound human premolars with no caries, restoration, enamel hypomineralization, cracks, or fluorosis in their buccal surface that had been extracted for orthodontic reasons. The study protocol was approved by the ethics committee of the university (IR.ABZUMS.REC.1401.121).

### Sample Size

The sample size was calculated to be 19 in each group according to studies by Farhadifard et al, [12] and Najafi et al, [13] assuming 95% confidence interval and study power of 80% using G Power software.

### Specimen Preparation

The teeth were cleaned by a scalpel and a toothbrush, and were stored in saline at room temperature until the experiment. They were immersed in 0.5% chloramine T solution one week prior to the onset of the erosion process. To induce enamel erosion, the teeth were immersed in 500 mL of Coca-Cola solution with a pH of 2.3 at room temperature for 2 minutes [7][8][10][14][15][16] and were then rinsed with water for 10 seconds. This process was repeated 3 times with fresh Coca-Cola solution. In other words, all teeth were immersed in Coca-Cola solution for a total of 8 minutes [10][14]. After completion of the erosive cycle, the specimens were stored in saline.

Prior to surface treatments, the buccal surface of all teeth was cleaned by a rubber cup. The teeth were then randomly assigned to 4 groups (n=19) for the following surface treatments [12]:

Group 1 (control): Acid etching alone: The buccal surface of the teeth was etched with 37% phosphoric acid (Etchant-37; Denfil, Korea) for 15 seconds, and then rinsed with water for 30 seconds.

Group 2: Bur grinding: The buccal surface of the teeth was ground by a tapered diamond bur with 1.2 mm diameter and 8 mm length with highspeed handpiece in two perpendicular directions under water spray.

Group 3: Sandblasting: The buccal surface of the teeth was sandblasted with 50 µm aluminum oxide particles with 65 psi pressure at 10 mm distance for 7 seconds using a sandblaster (Fineblast; Kpushafan Pars, China).

Group 4: The buccal surface of the teeth was subjected to Er:YAG laser irradiation (Fotona, China) with a SN614 laser handpiece with 2940 nm wavelength, 1.5 W power, 100 mJ energy density, 300 femtosecond pulse width and 15 Hz frequency in pulse mode [17]. The distance between the handpiece tip and the buccal surface of the teeth was 1 mm, and a cylindrical tip with 1.3 mm diameter was used. The air/water flow rate was 4 mL/s.

The teeth in groups 2-4 were then etched with 37% phosphoric acid as explained for group 1.

After the surface treatments, metal brackets (022 slot MBT American Orthodontics) were bonded to the buccal surface of the teeth using GC Ortho light-cure orthodontic adhesive (GC, Japan). For this purpose, the tooth surface and brackets were completely dried, composite was applied over the bracket base, and the bracket was positioned at the center of the buccal surface of the tooth. The bracket position was adjusted by using a dental explorer, and excess composite was removed. Composite curing was performed using a LED curing unit (Guilin woodpecker medical instrument Co., Ltd., Germany) in ortho mode. Light was irradiated directly to the brackets for 10 seconds, followed by 10 seconds of irradiation of the composite at the bracket-tooth interface from the left side, and 10 seconds from the right side. The specimens were then stored in saline.

### Thermocycling and SBS Testing

To better simulate the intraoral environment, the tooth-bracket assemblies underwent thermocycling for 3000 cycles in a thermocycler (TC300; Vafaei Industrial, Tehran, Iran) between 5-55°C with a dwell time of 20 seconds and a transfer time of 10 seconds [18]. After 48 hours, the specimens were mounted in auto-polymerizing acrylic resin (Acropars, Iran), and the SBS of brackets to the eroded enamel was measured in a universal testing machine (Zwick Roell, Ulm, Germany). Vertical load was applied to the enamel-bracket interface by a flat-end stainless steel blade parallel to the longitudinal tooth axis at a crosshead speed of 1 mm/minute until bracket debonding. To calculate the SBS in megapascals (MPa), the load required for bracket debonding in Newtons (N) was divided by the bracket base surface area in square-millimeters (mm2). The length and width of brackets were initially measured by a caliper to be 3.7 mm and 2.9 mm, respectively. Accordingly, the bracket base surface area was calculated.

### Determination of Adhesive Remnant Index (ARI) Score

**Figure-1:**
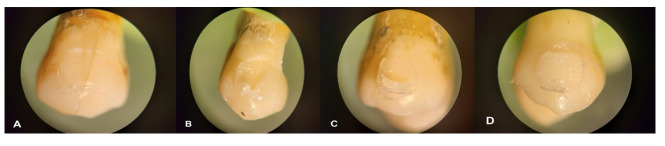
Figure 1. ARI scores; (A) score 0 (no adhesive remaining on the enamel surface), (B) score 1 (less than 50% of adhesive remaining on the enamel surface), (C) score 2 (more than 50% of adhesive remaining on the enamel surface), (D) score 3 (all adhesive remaining on the enamel surface)

After bracket debonding, the buccal surface of the teeth was inspected under a stereomicroscope (SMZ800, Nikon, Japan) at x10 magnification, and the ARI score was determined according to Artun and Bergland [16] as follows (Figure-1): Score 0 indicates no adhesive remaining on the enamel surface; Score 1 indicates less than 50% of adhesive remaining on the enamel surface; Score 2 indicates more than 50% of adhesive remaining on the enamel surface; and Score 3 indicates all adhesive remaining on the enamel surface.

### Statistical Analysis

Based on the Reynolds, I. R. (1975) study [17], optimal value for SBS in samples enamel bonding is 6 to 8 MPa. We considered SBS lower than 6 as non-optimal. Normal distribution of data was confirmed by the Shapiro-Wilk test (P>0.05). Thus, comparisons were made by one-way ANOVA followed by pairwise comparisons with the Dunnett post hoc test. All statistical analyses were performed using SPSS version 26 (SPSS Inc., IL, USA) at 0.05 level of significance.

## Results

**Table:** Table1. Measures of central dispersion for the SBS (MPa) of metal brackets to eroded enamel

**Group**	**Mean**	**SD**	**Minimum**	**Maximum**	**P value **
**Acid etching**	21.77	10.7	5.93	47.14	
**Bur grinding**	18.46	6.6	4.13	28.07	0.35
**Sandblasting**	18.17	8	7.97	36.05	
**Er:YAG laser**	17.44	5.72	11.13	31.92	

**Table:** Table2. Frequency Distribution of SBS Optimal Status Across Treatment Groups Optimal Status

**Optimal Status **	**Acid etching**	**Bur grinding**	**Er:YAG laser**	**Sandblasting**
**Non-optimal**	2 (10.5%)	0 (0.0%)	0 (0.0%)	0 (0.0%)
**Optimal**	17 (89.5%)	19 (100.0%)	19 (100.0%)	19 (100.0%)

**Table:** Table3. Frequency Distribution of Different ARI Scores

**ARI score**		**Acid etching**	**Bur grinding**	**Sandblasting**	**Laser irradiation**
**0**	Number	7	5	4	3
	Percentage	8.36	26.3	21.1	15.8
**1**	Number	4	5	4	5
	Percentage	21.1	26.3	21.1	26.3
**2**	Number	3	4	2	2
	Percentage	15.8	21.1	10.5	10.5
**3**	Number	5	5	9	9
	Percentage	26.3	26.3	47.4	47.4

### SBS

The SBS values, measured in megapascals (MPa), were assessed to determine the bonding effectiveness of metal brackets to eroded enamel surfaces after different surface treatments. The mean SBS values for the groups were as follows: acid etching (21.77 ± 10.7 MPa), bur grinding (18.46 ± 6.6 MPa), sandblasting (18.17 ± 8.0 MPa), and Er:YAG laser irradiation (17.44 ± 5.72 MPa). The acid etching group exhibited the highest mean SBS, while the Er:YAG laser group showed the lowest.

The standard deviations indicate variability within each group, with the acid etching group showing the highest dispersion (SD=10.7 MPa) and the Er:YAG laser group the lowest (SD=5.72 MPa). The minimum and maximum SBS values further highlight the range of bonding strengths, with acid etching ranging from 5.93 to 47.14 MPa, bur grinding from 4.13 to 28.07 MPa, sandblasting from 7.97 to 36.05 MPa, and Er:YAG laser from 11.13 to 31.92 MPa.

Statistical analysis using one-way ANOVA revealed no significant differences in SBS among the four groups (P=0.35).

As shown in Table-2, a chi-square test of independence was conducted to examine the association between SBS optimal status (Optimal: SBS≥6 MPa; Non-optimal: SBS<6 MPa) and treatment groups. The test revealed no significant association between SBS optimal status and treatment group, χ² (3, N=76) = 6.16, P=.10, suggesting that the distribution of optimal and non-optimal SBS values does not significantly differ across the groups.

### ARI Score

The frequency distribution of ARI scores (Table-3), which indicate the amount of adhesive remaining on the enamel surface after bracket debonding, was analyzed across the four groups, and the results were statistically non-significant (P=0.82).

In the acid etching group, the most frequent ARI score was 3 (26.3%, n=5), indicating that all adhesive remained on the enamel surface in these cases. This group also had 8.36% (n=7) with an ARI score of 0 (no adhesive remaining), 21.1% (n=4) with a score of 1 (less than 50% adhesive remaining), and 15.8% (n=3) with a score of 2 (more than 50% adhesive remaining). The bur grinding group showed an even distribution for ARI scores 0, 1, and 3 (26.3% each, n=5), with 21.1% (n=4) for a score of 2. The sandblasting group had the highest frequency of ARI score 3 (47.4%, n=9), followed by equal frequencies for scores 0 and 1 (21.1% each, n=4), and the lowest frequency for score 2 (10.5%, n=2). Similarly, the Er:YAG laser group also had the highest frequency at ARI score 3 (47.4%, n=9), followed by score 1 (26.3%, n=5), and equal frequencies for scores 0 and 2 (15.8% and 10.5%, respectively, n=3 and n=2).

## Discussion

In our study, SBS values compared among groups, was not successful in determining most effective surface treatment for bonding metal brackets to eroded enamel. Sandblasting yielded the lowest SBS, indicating it may not be suitable for achieving reliable bond strength in this context. The high variability in the Er:YAG laser group suggests that its effectiveness may depend on specific parameters or operator technique, warranting further investigation. The ARI score distributions showed no significant differences among groups, with a trend toward higher adhesive retention on the enamel surface (ARI score 3) in the sandblasting and laser groups.

Er:YAG laser was used in the laser group in the present study, which is the most commonly used laser type for dental hard tissue ablation. It is mainly absorbed by water; however, it has sufficient energy density to cause photoacoustic effects and cavitation with minimal thermal damage [18]. Laser irradiation changes the physical and chemical properties of the enamel, and roughens the surface. It eliminates the prismless enamel from the tooth surface and exposes the enamel rods for adhesive bonding. The laser group showed the lowest SBS in the present study; however, it had no significant difference in SBS with other groups. This finding may be due to the fact that although laser irradiation increases the surface roughness, the created porosities on the surface are irregular and do not follow a homogenous pattern [19]. Laser irradiation creates cup-shaped depressions with no undercut, which cannot provide optimal mechanical retention; while, acid etching creates regular undercuts that result in formation of homogenous resin tags that increase the bond strength [19]. Also, thermal degeneration of collagen fibers caused by laser irradiation can decrease the bond strength to enamel, although enamel has only 0.5% collagen [20]. The present result in this regard was in line with the findings of Sallam and Arnout [21]. They showed that Er:YAG laser irradiation with 2940 nm wavelength, 1.5 W power, and 15 Hz frequency had no significant effect on SBS of brackets. Çokakoğlu et al. [22] reported similar results as well; however, they showed that Er:YAG laser irradiation increased the SBS when a 2-step self-etch adhesive was applied. A total-etch system was used for bonding of brackets to eroded enamel in the present study. The same results were reported by Lopes et al [23]. However, in contrast to the present results, Kiryk et al. [24] reported that Er:YAG laser irradiation of enamel surface followed by acid etching significantly increased the SBS of brackets to sound enamel. Difference between their results and the present findings may be due to the fact that they evaluated bonding to sound enamel; whereas, eroded enamel was evaluated in the present study. Also, the laser parameters were different in the two studies. Different results were also reported by Najafi et al, [14] who showed that Er:YAG laser irradiation and acid-etching of bleached and desensitized enamel significantly increased the SBS to metal brackets. Difference between their results and the present findings may be due to evaluation of different types of enamel (bleached versus eroded enamel) and different laser parameters.

Bur grinding was also performed in the present study, which did not significantly change the SBS compared with other groups. Grinding eliminates the prismless enamel, which has lower potential for bonding, and exposes the enamel with higher bonding potential [25]. Enamel removal with grinding is minimal and limited to the 30-µm prismless enamel. It does not damage the tooth surface. The present results regarding no significant effect of bur grinding on SBS were in line with the findings of Najafi et al, [10] who found no significant difference between grinding and laser irradiation. However, grinding yielded a higher SBS than acid etching, and lower SBS than sandblasting in their study, which was different from the present results. Also, Farhadifard et al. [9] reported that grinding increased the SBS of old composite to ceramic brackets. Their results were different from the present findings due to evaluation of different bracket types, substrates, and adhesives. Moreover, the grinding parameters were not the same in the two studies.

In the present study, sandblasting could not significantly change the SBS compared with other groups. This result can be due to dispersion of alumina particles in the porous surface, preventing optimal penetration of adhesive into the porosities. Resultantly, the sandblasted surface cannot enhance the bond strength [26]. Also, sandblasting may roughen a surface larger than the bracket bonding area, which is another drawback [26]. Sandblasting increases the surface roughness and the available surface area for bonding [26]. The present result regarding sandblasting was in line with the findings of Oskoee et al, [27] although they compared Er,Cr:YSGG laser and sandblasting for enhancement of SBS of stainless-steel brackets to amalgam surfaces. Similarly, Lopes et al. [23] found no significant difference between Nd:YAG laser and sandblasting for enhancement of SBS of brackets to sound enamel. Nonetheless, Frhadifard et al. [9] reported that sandblasting significantly increased the SBS of ceramic brackets to old composite, which can be due to differences in bracket type, dental substrate, adhesive type, and sandblasting parameters between the two studies. Also, Najafi et al. [10] reported that sandblasting significantly increased the SBS of metal brackets to old composite. Difference between their results and the present findings can be attributed to evaluation of different bonding substrates.

In the present study, acid etching alone yielded the highest SBS, although it had no significant difference with SBS in other groups. Acid etching irregularly changes the enamel surface, and increases the surface free energy. Application of a resin-based liquid over this surface results in resin penetration into the surface irregularities and micro-mechanical interlocking following polymerization. The resin micro-tags formed within the enamel surface are the main mechanism of resin adhesion to the enamel [8]. The present results regarding the SBS of the acid-etched group were similar to the findings of de Vasconcelos Leão et al, [6]; nonetheless, Najafi et al. [10] reported that the acid-etched group yielded the lowest SBS, which was in contrast to the present findings. This difference may be explained by the difference in the type of substrate (eroded enamel in the present study versus old composite in their study).

Assessment of ARI scores revealed no significant difference among the four groups in the present study. Ideally, debonding should occur at the adhesive-bracket interface, and the outermost enamel surface should remain intact. Debonding at the adhesive-bracket interface minimizes the risk of enamel damage, and is therefore preferred by most orthodontists. However, debonding at the adhesive-bracket interface leaves higher amounts of residual adhesive on the enamel surface, which should be eliminated by bur. Debonding at the enamel-adhesive interface may damage the enamel surface, and is not favored by orthodontists [28]. An ARI score 0 indicates poor bonding at the enamel-adhesive interface while a score 3 indicates a strong bond at the enamel-adhesive interface [29] Although the present results showed no significant difference among the four groups in the ARI scores, sandblasted and laser-irradiated groups showed the highest frequency of ARI score 3.

The current results were in agreement with the findings of Najafi et al, [10] who showed no significant effect of CO2 laser irradiation of old composite on ARI score after metal bracket debonding. However, grinding and sandblasting increased the ARI score in their study, which was different from the present results. Different results were also reported by de Vasconcelos Leão et al, [6] who demonstrated that sandblasting (75 psi, 4 seconds, 10 mm distance) resulted in a higher ARI score compared with the non-sandblasted control group. Difference in the results may be attributed to different sandblasting parameters. Also, the adopted technique for induction of enamel erosion was different in the two studies. Moreover, the acid-etched control group in their study did not undergo erosion, which was different from the methodology of the present study. Furthermore, different adhesive types were used in the two studies. Nonetheless, it should be noted that the ARI is a subjective index, and experience and expertise of the clinicians can affect their judgment. This factor may also explain variations in the reported results in the literature [16]. Differences in the morphology of brackets, interfacial properties of the bracket-adhesive assembly, and thickness of the adhesive layer, which is influenced by the bracket base design, can also affect the results [30].

This study had some limitations. Despite the conduction of thermocycling, clinical environment cannot be perfectly simulated in vitro, which limits the generalizability of the findings. Also, only one type of laser with certain exposure parameters was used in the present study. Future studies are required on different laser types with various parameters, and also on sandblasting with different parameters in terms of particle size and pressure. Also, other adhesive systems and non-metal brackets should be assessed in future studies. Furthermore, changes in the enamel surface and hardness after erosion and surface treatments should be further evaluated. Finally, clinical studies are required to obtain more reliable results.

## Conclusion

Bur grinding, sandblasting, and Er:YAG laser irradiation did not significantly change the SBS of metal brackets to eroded enamel compared with acid etching alone, and all the tested methods yielded acceptable SBS values.

## Conflict of Interest

The author declares that they have no competing interests.
